# Comparative context analysis of codon pairs on an ORFeome scale

**DOI:** 10.1186/gb-2005-6-3-r28

**Published:** 2005-02-15

**Authors:** Gabriela Moura, Miguel Pinheiro, Raquel Silva, Isabel Miranda, Vera Afreixo, Gaspar Dias, Adelaide Freitas, José L Oliveira, Manuel AS Santos

**Affiliations:** 1 Centre for Cell Biology, Department of Biology, University of Aveiro, 3810-193 Aveiro, Portugal; 2 Institute of Electronics and Telematics Engineering, University of Aveiro, 3810-193 Aveiro, Portugal; 3 Department of Mathematics, University of Aveiro, 3810-193 Aveiro, Portugal

## Abstract

Codon context is an important feature of gene primary structure that modulates mRNA decoding accuracy. We have developed an analytical software package and a graphical interface for comparative codon context analysis of all the open reading frames in a genome (the ORFeome). Using the complete ORFeome sequences of *Saccharomyces cerevisiae*, *Schizosaccharomyces pombe*, *Candida albicans *and *Escherichia coli*, we show that this methodology permits large-scale codon context comparisons and provides new insight on the rules that govern the evolution of codon-pair context.

## Abstract 2

We have developed a system for comparative codon context analysis of open reading frames in whole genomes, providing insights into the rules that govern the evolution of codon-pair context.

## Background

The standard genetic code uses 64 codons for only 22 amino acids, including the amino acids selenocysteine and pyrrolysine whose incorporation into protein requires the reassignment of the UGA and UAG stop codons, respectively [1,2]. This degeneracy of the genetic code has important implications for gene primary structure evolution as it provides nature with a vast array of options for building open reading frame (ORF) sequences for any particular protein. However, the usage of synonymous codons for building ORFs is not random, suggesting the existence of mechanistic or evolutionary constraints that limit the degree of freedom for coding sequence building [3-6]. In other words, each organism uses a set of rules for building ORF sequences which restrict the total number of options provided by the degeneracy of the genetic code. These rules are only partly understood. Nevertheless, it is becoming increasingly clear that codon usage and context bias reflect the action of two main evolutionary forces: selection for mRNA decoding efficiency and mutational drift acting indiscriminately on coding and noncoding DNA [7-10].

Codon usage reflects selection for translational efficiency, as highly expressed genes tend to use codons that are decoded by abundant cognate tRNAs [11-13]. Similarly, the context of a sequential pair of codons (codon-pair) is biased, but this bias is apparently linked more to decoding accuracy than to translational speed [14-17]. This suggests that the translational machinery is sensitive to the nature of the codon-pair present in the ribosome A and P decoding sites [16,18-20], raising the possibility that, like codon usage, codon context may also be species specific. This is supported by the fact that tRNA populations diverge in the number and abundance of tRNA isoacceptors for each codon family and also in the pattern of modified nucleosides in the tRNAs, which also affects mRNA decoding accuracy.

To shed new light on the overall pattern of codon context at the species level and evaluate how codon-pair context varies between species, we have developed software and statistical methodologies for codon-pair context analysis on all the ORFs in a genome as a whole (the ORFeome). Because our main interest is to evaluate the effect of codon context on mRNA decoding accuracy, this study focuses on the context of codon-pairs and not on long-range context effects. With a few exceptions, long-range context is not relevant for mRNA decoding by the ribosome. These new methodologies were tested using the complete ORFeome sequences of the eukaryotes *Saccharomyces cerevisiae*, *Candida albicans *and *Schizosaccharomyces pombe *and the bacterium *Escherichia coli*. The methodology developed provides robust and flexible tools for intra- and inter-ORFeome comparative codon-pair context analysis, permits identification of species-specific codon context fingerprints and provides new insight into the role of codon context on mRNA decoding accuracy and ultimately on the pressure imposed by the translational machinery on the evolution of the ORFeome. The software developed, called Anaconda, is available at [21].

## Results

### Global analysis of codon context in yeast

**Figure 1:**
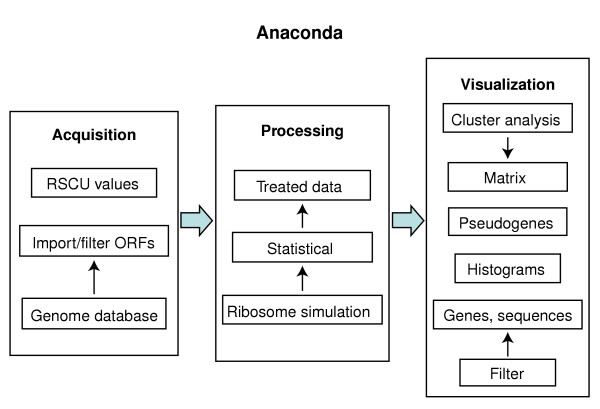
Architecture of the Anaconda bioinformation system. The Anaconda package contains a data-acquisition module that permits downloading raw data from genome databases and filter it into a local database. This data is then processed using a ribosome simulation algorithm and transferred to a 64 × 64 table that renders itself to statistical analysis. The processed data is then transferred to the visualization module that has a number of different tools that permit different types of data visualization and analysis. RSCU, relative synonymous codon usage values from very highly expressed genes, necessary for codon adaptation index (CAI) calculation (see [55]).

The Anaconda bioinformatics system developed in this study identifies the start codon of an ORF and reads it by moving a 'decoding window' three nucleotides at a time in the 3' direction until it encounters a stop codon. While doing so it fixes the middle codon of the reading window and memorizes its 5' and 3' neighbors. Anaconda creates a table of frequencies of 64 × 64 codons that allows computation of the number of times the complete set of contiguous codon pairs occurs in an ORF or in an ORFeome. The overall architecture of Anaconda is described in Figure 1.

**Figure 2:**
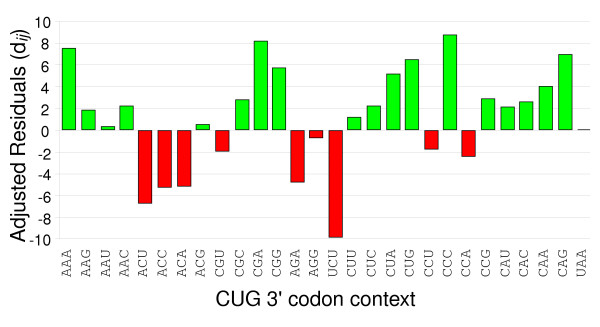
Codon context is highly biased in yeast. The bar chart shows the distribution of the adjusted residual values given in Table 1 for the 3' context of the *S. cerevisiae *CUG codon. See Table 1 legend for details.

**Figure 3:**
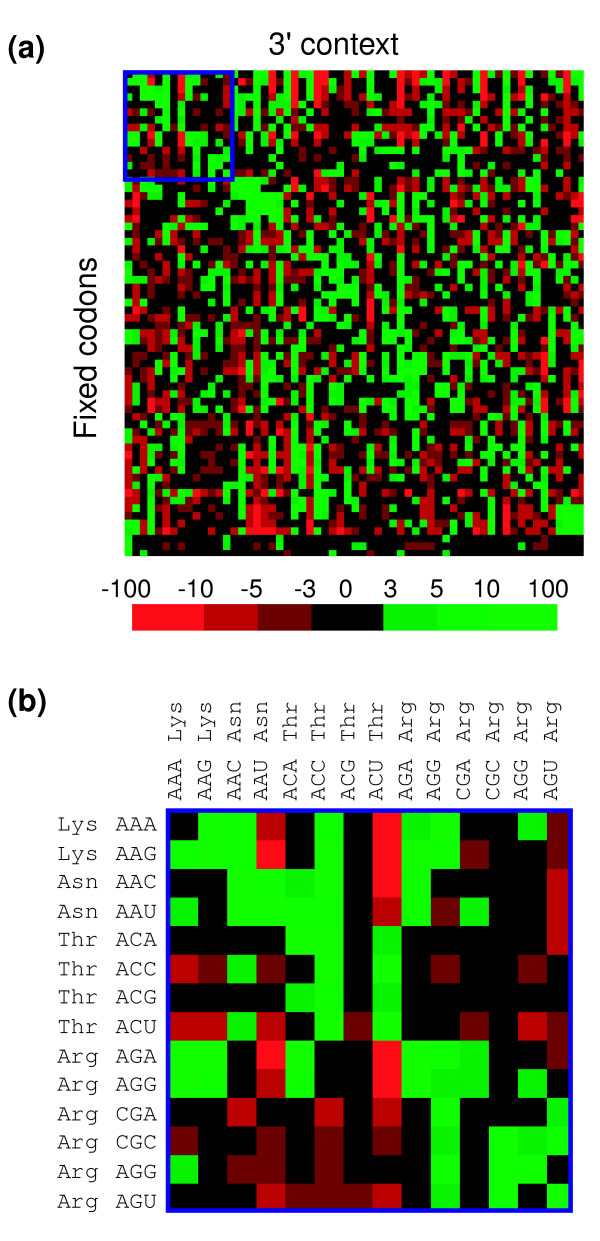
*S. cerevisiae *genome map of codon context. For visualization purposes the values of the residuals of the 64 × 64 codon context table were converted into a color-coded map in which red represents the negative values (bad context) and green the positive values (good context). The values that are not statistically significant are indicated in black (-3 to +3). The color scale represents the full range of values of residuals for yeast codon context. Fixed codons represent the P-site codons and the 3' context refers to the A-site codons as viewed by the ribosome simulation software module. **(a) **The yeast complete 3' codon context map shows a diagonal green line, which indicates that most codons prefer themselves as neighbors on their 3' side. The map also indicates that without exception, each codon prefers a defined set of neighbors (green) and avoids others (red). The intensity of red and green indicates the extent of the preference or rejection. **(b) **Codons that are represented in the map can be visualized by zooming into particular areas of the map (boxed in dark blue in (a)). The order of the fixed and 3' context codons indicated in (b) is predefined in the software module.

**Figure 4:**
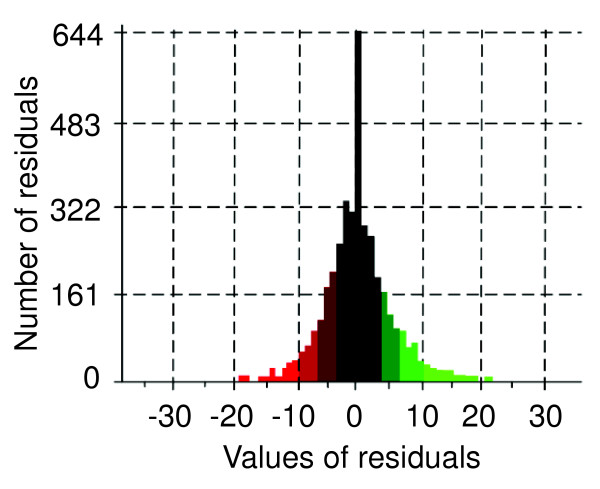
Distribution of the adjusted residuals from the *S. cerevisiae *codon context map. Forty-three percent of the residuals fall within the nonsignificant -3 to +3 interval, indicating that a very large number of codon combinations are not significant to the rejection of independence - that is, are not significantly preferred or rejected in this genome.

The codon-pair context frequency table built by Anaconda allows the statistical analysis of contingency tables to be used to test whether the context is significantly biased [22-25]. These tables allow one to test the existence of association between codon-pairs through the chi-square (χ^2^) test of independence; to identify preferred and rejected pairs of codons in the ORFeome through the analysis of adjusted residuals for contingency tables (Table 1 and Figure 2); and to construct a codon context map on an ORFeome scale (Figure 3). The Anaconda algorithm, its graphical interface and implemented statistical methodologies were tested using the yeast *S. cerevisiae *ORFeome. For this, the complete ORFeome was downloaded from the yeast genome database [26], the adjusted residual values for the total number of codon pairs were calculated (see Materials and methods) and each residual value present in a cell of the contingency table (64 lines × 64 columns) was converted into a two-color coded map (Figure 3). In the latter, green represents positive values greater than +3 (herein called preferred codon-pairs) and red represents negative values lower than -3 (herein called rejected codon-pairs) according to the color scale indicated in Figure 3a. The data clearly show that each codon has a set of preferred 3' codon neighbors (green) and rejects a set of other codons (red), indicating that codon context is highly biased in *S. cerevisiae*. However, in a rather large number of cases, the 3' codon context is not biased or at least strongly rejected or preferred. This is indicated by the black color in the map (Figure 3) and in the histogram of the residuals distribution (Figure 4). This black color corresponds to residual values that fall within the interval of -3 to +3 and correspond to codon contexts that do not contribute to the bias for a confidence level of 99.73% (Table 1 and Figure 2). The overall empirical distribution of residual values for codon context in the yeast ORFeome (Figure 4) clearly shows that a large fraction (about 47%) of codon-pair contexts fall within the interval of -3 to +3, indicating that in many cases the context may not be under high selective constraint.

### Codon clustering unveils unique features of codon context

**Figure 5:**
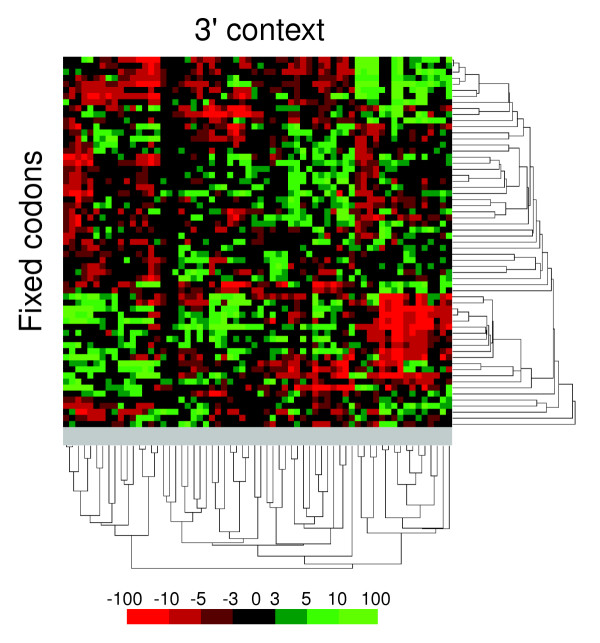
Codon context bias is organized in discrete groups. A two-way Pearson clustering by single linkage of the codon context data highlights regions of good and bad codon context, indicating that codon context bias is highly structured. A significant number of codons do not fall into the major clusters, indicating that their preferences and rejections are defined on a one-to-one basis. The 3' codon contexts whose residual values fall within the nonstatistically significant -3 to +3 interval are also scattered in the map, indicating that there is no cluster of codons that have little or no preference for particular codons as 3' neighbors.

The codon-pair context maps shown in Figure 3a,b were built using a manually predefined distribution of codons in both lines and columns. To better understand the full extent of the codon-pair context bias in yeast, the data were clustered using the Pearson's correlation coefficient [27], which enables grouping of codons with similar context preferences. Using double clustering (that is, clustering both lines and columns) several distinct groups of red and green codon-pair contexts were identified for the *S. cerevisiae *ORFeome, thus showing that certain groups of codons have similar 3'-neighbor preferences (Figure 5).

**Figure 6:**
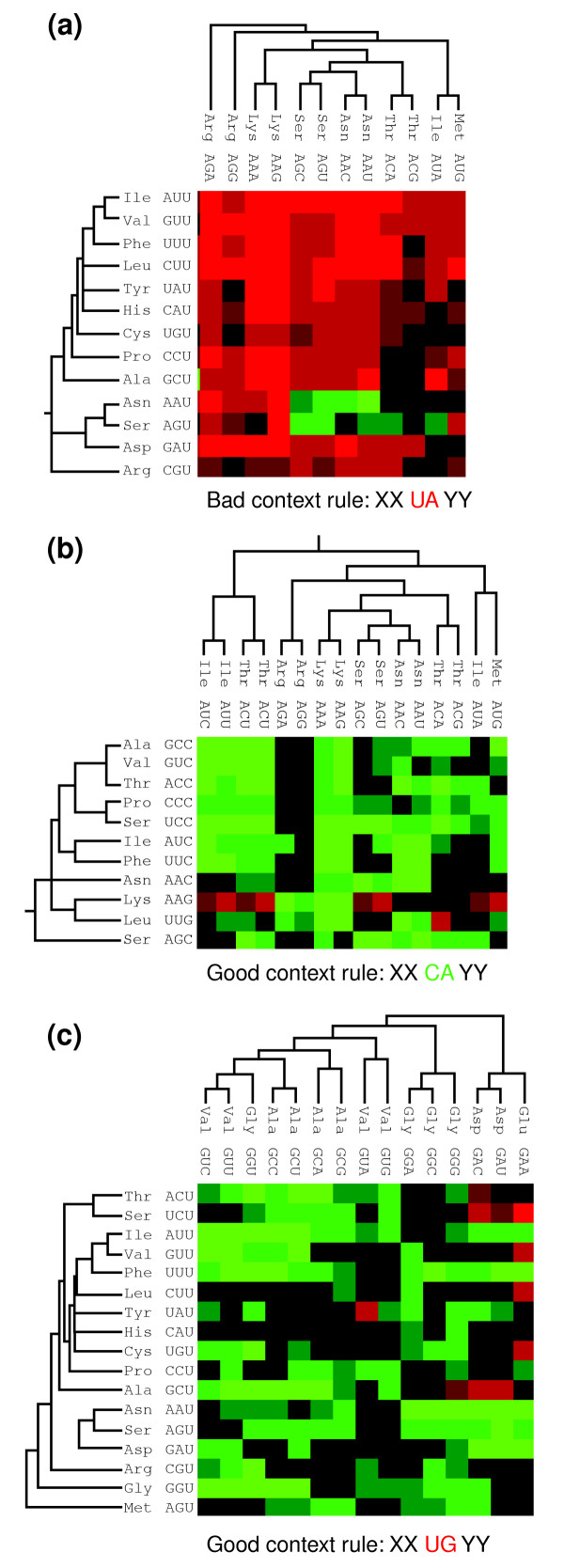
Codon clusters define specific codon-context rules in *S. cerevisiae*. **(a) **A major cluster of bad context is defined by codon pairs whose wobble base of the first codon is uridine (U) and the first base of the 3' neighbor is adenosine (A). This cluster defines a XXU-AYY context rule, in which X and Y are any nucleotide. Within this cluster some of the Asn and Ser codons represent exceptions to the above rule as their residual signal is positive (green cells). **(b,c) **Two of the good context clusters define two distinct codon context rules, namely (b) XXC-AYY and (c) XXU-GYY rules. As before, some of the codons within those clusters are exceptions to the above rules and a number of codons have no particular preferences or rejections (black cells).

To identify the codons responsible for defining the subgroups with high bias (red and green clusters) and evaluate whether these could define codon-pair context rules, one zooms in on the context subclusters. Three specific subclusters (one red and two green) were analyzed in this study (Figure 6a-c). The red subcluster shown in Figure 6a is defined by codon-pairs in which the last nucleotide of the first codon is uridine (U) and the first nucleotide of the next codon (3' side) is adenosine (A). As no such rule was observed for the other codon positions - that is, positions 1 and 2 or 2 and 3 of codon 1 or positions 1 and 2 or 2 and 3 of codon 2 (data not shown), the codons are clustered based on the following context rejection rule: XXU-AYY. The intensity of rejection (given by the adjusted residual itself) is not identical for all codon combinations within the subcluster. However, with the exception of the asparagine AAU and serine AGU codons, and some others whose residual values fall within the non-statistically significant -3 to +3 interval, all other U-ending codons avoid 3'-neighbor codons starting with an A. If one assumes that fixed codons in the map (lines) represent P-site codons and 3' codons (columns) represent A-site codons, then the above rule suggests that the third base of a P-site codon somehow influences the choice of the first base of the A-site codon. In other words, and assuming that context modulates decoding accuracy, *S. cerevisiae *codon pairs that end with an U and start with an A are likely to cause some trouble to the ribosome during decoding.

The above observations were confirmed by analyzing two green codon-pair context subclusters (good contexts). In these cases, two different clustering rules were identified, namely the XXC-AYY and the XXU-GYY (Figure 6b,c). Like the bad context subcluster discussed previously, in these good context subclusters there are exceptions that include red and black context cells. Nevertheless, there is a strong trend for the above rule within each subcluster, indicating once more that the third base of the P-site codon influences the first base of the A-site codon. The fact that these rules cannot be seen for other codon positions, and that there are exceptions to these rules for other codon families in the overall map, excludes the possibility that the third-first base rules identified reflect dinucleotide preferences or rejections arising from DNA replication and repair ([28] and see later).

### Comparative codon context analysis

**Figure 7:**
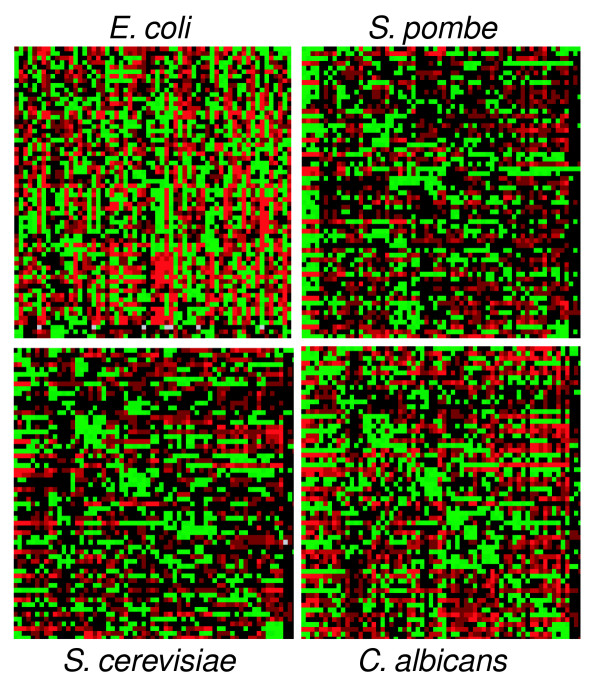
Codon context maps are species specific. Comparison of the genomic codon context maps of *S. cerevisiae*, *C. albicans*, *S. pombe *and *E. coli *shows that they are all different. There are common features between the maps but differences are clearly visible, indicating that each species has a specific set of codon context rules. Among the common features, the green diagonal line in the yeast maps is the most relevant. This diagonal indicates that almost all codons prefer themselves as their 3' neighbors and is strongly marked in the *C. albicans *context map, suggesting that in this species, tandem codon repetition is very common.

Because the *S. cerevisiae *codon-pair context map produced a clear context pattern, we wondered whether this map could represent a species-specific fingerprint, as is the case for the codon-usage fingerprint. For this, maps for *S. pombe*, *C. albicans *and *E. coli *were also constructed, with the latter being used as an outgroup. Some similarities between the codon-pair context maps were immediately visible, namely a strong green diagonal line in the yeast maps (Figure 7). There are, however, important differences that become evident when the negative and positive residual values are ranked for the yeast species studied (Table 2). These values represent the most negative and positive residuals of the yeast maps and consequently provide a good indication of the differences in codon context present in the three yeast species. Of the 10 most positive residual values ranked in Table 2, only two are common for the three yeast species, namely GAA-GAA, GGU-GGU and GCU-GCU. A similar result was obtained when the most negative values were ranked (Table 2). In addition, the *C. albicans *genome shows a more biased codon-pair context status. For example, the 10th most positive residual (49,476 for ACA-ACA) is higher than the maximum residual value for *S. cerevisiae *and *S. pombe*: 45,422 for CAG-CAG and 35,086 for UCU-UCU, respectively (Table 2).

**Figure 8:**
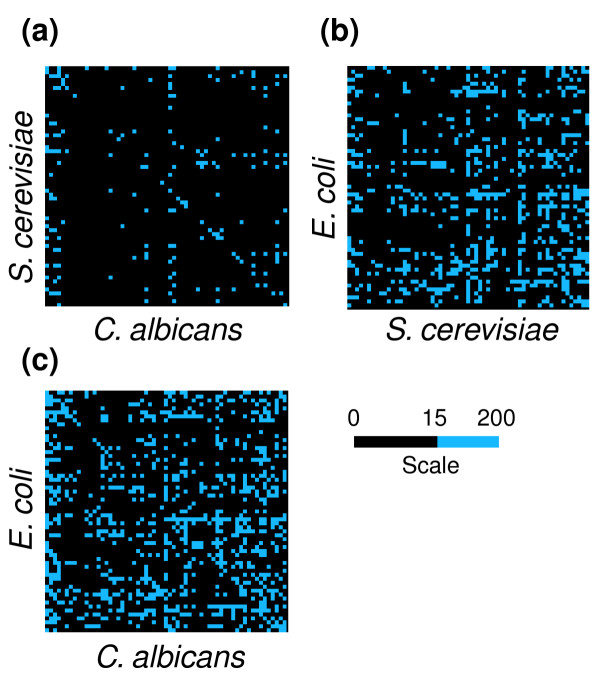
Differential display maps for comparative analysis of codon context. To compare the codon context maps of different species, the order of the codons displayed in the map was fixed and the maps overlapped using a differential display tool built into the Anaconda bioinformation system. Maps representing the context differences between **(a) ***S. cerevisiae *and *C. albicans*, **(b) ***E. coli *and *S. cerevisiae *and **(c) ***C. albicans *and *S. cerevisiae *were obtained by calculating the module of the difference between the residuals of each map. The differences are represented in blue according to the color scale. The blue cells indicate the highest context difference and the black cells represent pairs of codons that have similar residual values between two species (module of the difference between residuals falls within the 0-15 interval). The maps show rather large differences in codon context between *E. coli *and *S. cerevisiae *or *C. albicans *and smaller differences between *S. cerevisiae *and *C. albicans*.

An additional approach to identifying codon-pair context differences between *S. cerevisiae*, *S. pombe *and *C. albicans*, was undertaken by overlapping the complete codon context maps displayed in Figure 7. For this, the maps built with a predefined order of codons for both the 64 lines and the 64 columns were merged, allowing the construction of a comparison codon-pair context map. We call this a differential codon-pair context map (DCM) and it corresponds to the module of the difference between the residuals of overlapped cells of the 64 × 64 context table (Figure 8). A new color scale based on gradation of blue was used for the differential display. Using this methodology, the codon context differences for the three yeast species became self-evident, indicating that codon context - like codon usage - is species specific (Figure 8). In all three DCMs shown in Figure 8 there are common features, which are indicated by the black cells; however, the differences (blue) are clearly visible. As expected from the phylogenetic distance of the various species studied, the DCMs for the pairs *E. coli*-*S. cerevisiae *and *E. coli*-*C. albicans *show many more differences than the DCM for the pair *S. cerevisiae*-*C. albicans*.

The DCMs also show that codon-pair context is more similar for the pair *S. pombe*-*S. cerevisiae *(data not shown) than for the other two yeast pairs, indicating that there are fewer differences between *S. pombe *and *S. cerevisiae *than between *C. albicans *and *S. cerevisiae*. This is surprising, considering that *S. pombe *diverged from *S. cerevisiae *420 million years ago whereas *C. albicans *diverged from the latter only 170 million years ago [29]. The effect of the rather strong green diagonal (codon repeats) in the *C. albicans *maps is also visible in the DCMs (blue cells) of the *C. albicans*-*S. cerevisiae *pairs (Figure 8a). In order to shed more light on the differences in the codon context maps for the three yeasts, codon pairs were ranked according to the module of the difference between residuals (Table 3). Surprisingly, only one codon pair for the three yeast species (CAA-CAA) is present among the 10 highest values that were ranked. Further, the difference between these three species is not only qualitative, as shown above, but is also quantitative. For example, for the *S. pombe-S. cerevisiae *pair, the highest difference was found for the pair CAG-CAG with a value of 27,798, whereas in the *S. pombe-C. albicans *map the CAA-CAA pair showed a difference value of 100,639. In fact, in the latter yeast pair DCM all 10 values related are higher than the highest value (27,798) found for the CAG-CAG codon pair in the *S. pombe*-*S. cerevisiae *map (Table 3). Therefore, when taken together, DCMs and residuals rankings provide unique insight into the codon-pair context differences, even for phylogenetically related species such as yeasts.

### Contribution of mutation bias to codon-pair context

An important feature of the codon-pair context map in the yeasts analyzed, but not in *E. coli*, is the presence of a diagonal green line (Figures 3, 7). The existence of this green line implies that in those yeasts, most codons prefer to have another identical codon on their 3' side, indicating a degree of tandem codon duplication in the ORFeome of yeasts. Trinucleotide repeats are characteristic of eukaryotic genomes and have been attributed to DNA polymerase slippage during genome replication [30]. Whether the codon duplication observed in the ORFeome of the yeasts analyzed is a consequence of DNA replication only, or also reflects an evolutionary constraint imposed by the mRNA decoding machinery on those ORFeomes, is not yet clear and we are currently investigating this. In any case, this diagonal line in the codon context maps of yeasts is a strong feature, since the highest residuals of codon pairs (preferred pairs) occur for tandem codon repeats (Table 2).

**Figure 9:**
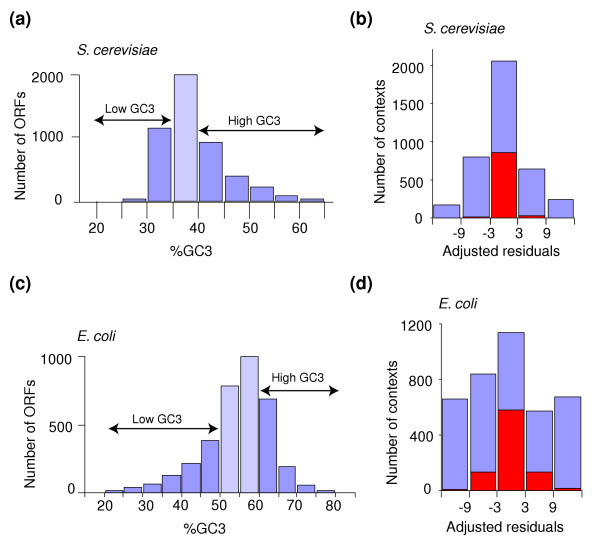
GC3 distribution in the complete ORFeome of *S. cerevisiae *and *E. coli *and its influence on the overall codon-pair context analysis. In order to study the role of mutational bias upon codon-pair context the ORFeomes of both **(a,b) ***S. cerevisiae *and **(c,d) ***E. coli *were distributed according to the %GC3 of individual ORFs. The GC3 of the *S. cerevisiae *and *E. coli *ORFeomes varied between the intervals 11.9-76.7% and 20-89.4%, respectively. For *S. cerevisiae*, however, most ORFs had a %GC3 between 35 and 40% (light blue bar in (a)), while for *E. coli *the majority of the ORFs have a %GC3 between 50 and 60% (light blue bars in (c)). Determination of the codon-pair context for the low and high GC3 subgroups permitted identification of their context differences. The computation of the number of residuals that changed their signal (for example, positive to negative) from one subgroup (low GC3) into the other (high GC3) provided a quantitative measure of the role of GC3 on codon-pair context (red bars in (b) and (d)). For both *S. cerevisiae *and *E. coli *GC3 bias has a strong effect on codon-pair context for weak residuals (-3 to +3), but no such effect was observed for contexts with the highest residuals (strong context), indicating that GC3 bias is mainly felt in weak codon-pair contexts.

**Figure 10:**
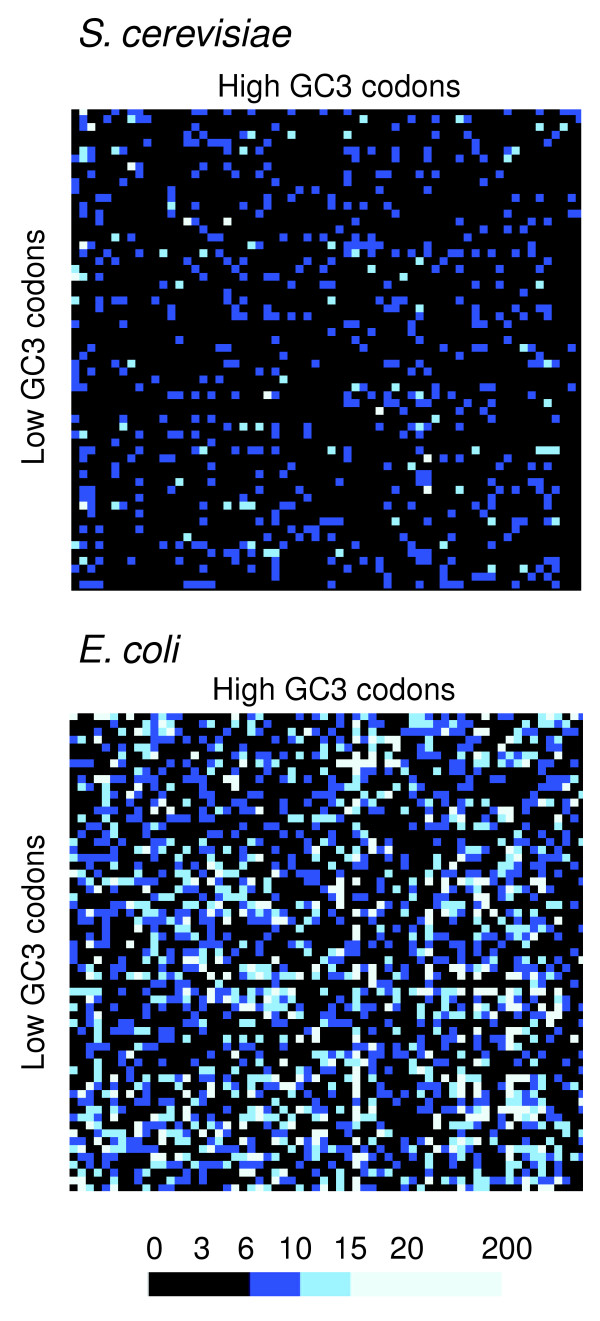
ORFs with low and high GC3 have different codon-pair contexts. To highlight the effect of GC3 bias on codon-pair context, the context maps for the subgroups of low GC3 and high GC3 ORFs of both *S. cerevisiae *and *E. coli *were overlapped using the differential display codon-pair context (DCM) tool. The DCM maps for *S. cerevisiae *and *E. coli *showed significant differences (light blue cells in the DCMs), in particular in *E. coli*, indicating that GC3 bias influences codon-pair context.

The above observations prompted us to investigate whether mutational bias also played a part in codon-pair context bias and whether such bias could be extracted from the codon-pair context maps. For this, particular attention was given to GC content because it plays a major role in codon usage [31]. An algorithm was implemented into Anaconda for calculating %GC total, %GC at codon position 1 (GC1), %GC at codon position 2 (GC2) and %GC at codon position 3 (GC3). While scanning an ORFeome, Anaconda divides ORFs into GC-content subgroups and creates groups of ORFs with high and low GC content. It also determines the distribution of ORFs according to their GC total and GC3 (Figure 9a,c). Codon-pair codon context maps can be built for each subgroup of codons and the maps compared using the DCM tool (Figures 9b,d and 10).

Because GC bias is better observed at the third codon position as a result of the degeneracy of the genetic code, GC3 was used to evaluate whether mutational bias contributed to the codon-pair context using the *S. cerevisiae *and *E. coli *ORFeomes as proof of principle. In the former, the ORF distribution varied from a minimum of 11.9% to a maximum of 76.7%; however, most ORFs fell within a narrow interval between 35-40% GC3 (Figure 9a). In the case of *E. coli*, the ORF distribution is broader, varying from a minimum of 20.0% to a maximum of 89.4%, but most ORFs have a GC3 between 50% and 60% (Figure 9c). This distribution made it possible to build codon-pair context maps for the low GC3 and high GC3 subgroups. As differences between these low and high GC3 context maps were expected to allow for evaluation of the importance of the bias introduced by mutational drift into the codon-pair context maps, these maps were overlapped using the DCM tool. As before, the maps were built using a single colour (blue) to aid visualization of the context differences. If mutational drift did not contribute to the context bias, the codon-pair context maps of the GC3 subgroups would be identical, producing a black differential display map. This is because the difference of the module of the residuals would be zero for all cells of the table of residuals.

The differential display map for the low and high GC3 ORF subgroups of *S. cerevisiae *showed several differences, indicating that GC bias contributes to the codon-pair context. However, most of these differences corresponded to small deviations in the strength of the rejection or preference of the codon-pair contexts (Figure 9b and 10, see also Table 4). In other words, the residual values had the same positive or negative signal in both cases but the value was higher in one GC3 subgroup than the other and vice versa. In some cases, an inversion of signal of the residuals (for example, from positive to negative) was detected, indicating that the residual of the codon-pair was positive in one GC3 subgroup and negative in the other GC3 subgroup (light blue in Figure 9b). This inversion of signal provides clear evidence for the influence of GC content bias in the codon-pair context. Similar results were obtained for the *E. coli *ORFeome; however, a much larger number of inversions of the residual signal was observed in this case, indicating that the GC content bias is far stronger in *E. coli *than in *S. cerevisiae *(Figures 9d and 10, see also Table 4). The reasons for these differences and the quantitative contribution of mutational bias to codon-pair context bias is not yet fully understood and is currently being investigated. However, Anaconda already provides strong evidence for a role for mutational bias on codon-pair context.

## Discussion

Codon context has been extensively studied in prokaryotic, eukaryotic, mitochondrial and viral genomes, and these studies unequivocally showed that codon-pair context is biased [9,10,32-35]. However, no tool has yet been developed to display codon context data and in particular codon-pair context (short-range context) in a way that would facilitate interpretation of the data and allow inter- or intra-genome context comparisons. This is essential if putative general rules that govern codon-pair context evolution are to be unraveled. The Anaconda bioinformation system has been developed to address this problem. By using statistical methodologies based on contingency tables and residual analysis (see Materials and methods), specific codon-pair context patterns were unveiled and displayed using a color coded ORFeome-context map. The data highlighted codon-pair context bias in yeasts and *E. coli *and some rules that define codon-pair context patterns in yeast.

### Forces that shape codon-pair context

Studies carried out in the 1980 s in *E. coli *have demonstrated that codon-pair context influences mRNA decoding accuracy and efficiency, indicating that the translational machinery imposes significant constraints on codon-pair context [17,36,37]. For example, in starved *E. coli *cells, the asparagine AAU and AAC codons are misread as lysine at high frequency [16]. Quantification of the level of lysine misincorporation at those codons and determination of the effect of the 3' nucleotide context on lysine misincorporation showed that the AAU codon is misread up to nine times more frequently than the AAC codon, and that the 3' nucleotide context (III-I context) influenced the level of misreading by as much as twofold [16]. Additional studies carried out *in vitro *in *E. coli*, have also shown that ribosomes discriminate C-ending Phe UUC and Leu CUC codons less well than the U-ending Phe UUU and Leu CUU, showing that synonymous codons differ in translational accuracy [38]. Therefore, a possible role for codon-pair context is minimization of decoding error, in particular in those codons that are poorly discriminated by the ribosome.

In *E. coli*, over-represented codon-pairs are translated more slowly than under-represented codon-pairs, indicating that codon-pair context also influences translational speed [14]. This suggests that codon-pair context in *E. coli *is under strong selective constraints imposed by the translational machinery. Whether the context patterns now unveiled in yeast reflect similar selective constraints remains unclear. Nevertheless, the codon-pair context maps described here provide a good starting point to address this important biological question *in vivo *in yeast in a guided manner. Additional evidence for a role for selection on codon-pair context was highlighted by the negligible, or even zero, contribution of GC3 to the context bias in very frequent or very infrequent codon-pairs (strong contexts) in both *S. cerevisiae *and *E. coli *(Figure 9, Table 4) and by a number of exceptions to the context rules that define the subclusters of codon-pairs (Figure 6). For example, within the XXU-AYY subcluster of rejected codons (Figure 6a), the codon pairs AAU-AGC, AAU-AGU, AAU-AAU, AAU-AAC and the set of AGU-AGC, AGU-AGU, AGU-AAU, AGU-ACA, AGU-AUA have positive residuals, indicating that they are codon pairs preferred by the ORFeome. Similar exceptions are found within the subclusters of preferred codon pairs shown (Figure 6b,c). Furthermore, a detailed analysis of the overall ORFeome context map (Figure 5) shows that other codon-pairs violate the XXU-AYY rules, namely GGU-AUG, GGU-AUC, GGU-AUU, GGU-ACC, GGU-ACU. This supports the hypothesis that those clusters of the context map are not formed on the basis of particular dinucleotide combinations that may be related to genome mutational drift. This is further confirmed by our observation that the dinucleotide preference in the XXU-AYY, XXC-AYY and XXU-GYY codon pairs is not observed when the various positions within each codon or codon-pair are analyzed. In other words, in the codon pair X_1_X_2_X_3_-Y_1_Y_2_Y_3_, the X_3_-Y_1 _preferences are not observed for the dinucleotides X_1_-X_2_, X_2_-X_3_, Y_1_-Y_2 _and Y_2_-Y_3 _(data not shown).

Despite these arguments, mutational bias does influence codon-pair context [7,39-41]. Observed mutational bias reflects mutational events that act indiscriminately on all DNA sequences (coding and noncoding DNA) and is consequently a property of the genome rather than the result of selection acting within ORFs [42-45]. The data presented here is in line with those observations. For example, context maps shown in this study indicate that several of the context clusters are formed on the basis of dinucleotide context rules (III-I rule), namely the XXU-AYY, XXC-AYY, XXU-GYY (Figure 6a-c). As dinucleotide context is related to DNA repair and replication constraints those clusters reflect mutational bias [28]. An important feature that highlights the influence of mutational bias on codon-pair context is GC content, in particular GC3 content. GC content has a strong influence in codon usage and in extreme cases can even drive certain codons out of ORFeomes [46,47]. The data presented here clearly show that GC3 affects codon-pair context; however, this effect is mainly visible for codon-pairs that have weak residuals (Table 4, Figure 9). As strong residuals (either positive or negative) provide an indirect measure of the strength of the codon-pair association, it is likely that for extreme residuals GC3 bias introduces only noise into the analysis whereas for residuals near the statistically nonsignificant interval (-3, +3), GC3 bias represents a major contribution to the context bias observed (Figure 9).

Apart from those cases mentioned above, other species-specific genomic features also contribute to codon-pair context bias highlighted by Anaconda. For example, the yeast codon-pair context maps show a feature of eukaryotic genomes which is not related to mRNA translation: trinucleotide repeats which are evident in the diagonal line present in Figures 3 and 7. This strongly suggests that there is a very high degree of tandem codon repeats (trinucleotide repeats), which are likely to arise from biased DNA replication (DNA polymerase slippage, see [30]). Whether these repeated codon-pairs improve mRNA translation efficiency or accuracy in yeast remains to be determined experimentally. As far as we are aware, there is no experimental evidence showing increased decoding accuracy or efficiency at those sites.

Finally, constraints imposed by protein sequences and mRNA secondary structure are also thought to influence codon context [48,49]. The context maps seem to exclude the former hypothesis because no cluster is formed as a result of selection or rejection of two adjacent amino acids. In regard to the latter constraint, the Anaconda algorithm was not designed to detect mRNA secondary structures and consequently this question cannot be addressed at this stage.

## Conclusions

The Anaconda algorithm was developed with the aim of studying codon-pair context on an ORFeome scale, define rules that govern codon-pair context, carry out large-scale interspecies codon-pair context comparisons and clarify the effect of selection and mutational drift on codon-pair context. The results provide important new insight on the role of codon-pair context on mRNA decoding accuracy and efficiency, and we expect that it will allow the development of reporter genes for *in vivo *and *in vitro *quantification of codon-decoding error and translational speed. Finally, Anaconda will be a valuable tool to redesign ORFs for efficient and accurate heterologous or homologous protein expression in yeast and, eventually, in other suitable host systems.

## Materials and methods

### Statistics

To study the association between contiguous codon-pairs, the coding sequences analyzed by Anaconda are processed in a 64 × 64 contingency table subdivided in mutually exclusive categories. If the 3' context is being analyzed, the rows of the table correspond to the codons in the P-site and the columns to the codons in the A-site of the ribosome. At the 5' context analysis the situation is inverted, and so the contingency table built is a transposed version of the one for 3' analysis.

A number of different mathematical methodologies have already been used to study codon context bias (for example [9,50-52]). In this study, the analysis of contingency tables and residuals (Figure 3) was considered appropriate, assuming a multinomial probabilistic model for the contingency table (a detailed discussion of this model in the context of genomic data can be found in [53]). In general, all these methodologies are based on *z*-score-type tests and give information about preference and rejection. Basically, those methodologies differ in the probabilistic model assumed, leading to statistics whose probability distribution is in most cases unknown. The advantage of the methodology proposed here is that its theory of inference is well known, yielding an analysis that is more sequential, more easily interpretable and with more complementary tools for analysis (for example, measures of association). In other words, this methodology was chosen because the adjusted residual values give direct information about preference and rejection in relation to what would be expected on a random basis. Furthermore, the probability distribution under the hypothesis of independence is determined without data simulations.

For analysis of contingency tables and residuals [22-25], given an *r *× *c *contingency table where a multinomial distribution is assumed (Table 5), the hypothesis of independence between the variables A and B is tested using the Pearson's statistic given by:



where:



It is known that Pearson's statistic has an asymptotical chi-square probability distribution with (*r *- 1)(*c *- 1) degrees of freedom. To identify cells in the table responsible for the eventual rejections of independence, the adjusted residuals *d*_*ij *_are calculated by:



where:



is the variance estimated for *r*_*ij*_. Haberman [54] has shown that, under independence between A and B, the adjusted residuals *d*_*ij *_have a standardized normal probability distribution, and therefore *P*(- 3 <*d*_*ij *_< 3) ≈ 0.9973, as *N *→ + ∞. This means that, for a 99,73% confidence level, the pair (A_i_, B_j_) is considered responsible for rejection of the hypothesis of independence if |*d*_*ij*_| ≥ 3. In practice, we consider that an adjusted residual is statistically significant if its absolute value is greater then 3.

Additionally, to find codon context patterns in the contingency table, lines and columns can be grouped using classifying methodologies such as cluster analysis. These patterns are determined by calculating similarities between two vectors of the contingency table using the centred Pearson correlation coefficient and applying single linkage. The single-linkage method produces groups with 'chaining effect': that is, any element of a group is more 'similar' to an element of the same group than to any element of another group.

### Software

The architecture of the Anaconda software is based on three main modules, namely data acquisition, processing and visualization (Figure 1). Each module works independently from the others and can easily be replaced or updated. Also, this component-based approach allows for insertion of new modules or new tools in each module, such as new statistical features.

The acquisition and processing modules download row data from genome databases, create a local database of usable ORFs and analyze the data using an algorithm that simulates the ribosome during mRNA decoding. It finally constructs a database containing the processed data. This data is then submitted to statistical analysis as described above. The visualization module allows the user to visualize the data matrices and gene sequences and to create filters that permit searching for specific sequence patterns defined by the user.

The data-acquisition module deals with genome input files, namely reading and interpreting FASTA sequences of complete or partial sets of ORFs from public or private genome databases. To ensure that the screened sequences have the best possible quality, and hence do not introduce background noise in the following analyses, several quality filters are applied to the reading process. When the filters are activated the data are classified according to the following criteria. Valid data consist of genes whose sequence is a multiple of three; which start with an AUG codon and stop with a UAG, UAA or UGA codon, and which satisfy other user-defined requirements. Rejected data consist of genes whose sequence does not fulfill the above requirements. The result is the separation of valid from rejected ORFs. Other parameters needed by the application, such as reference relative synonymous codon usage (RSCU) values for codon adaptation index (CAI) calculation [55], are also uploaded by this module.

The processing module is the core of the application, where the codon context analysis is performed. After prescanning the files, the user can test the existence of significant bias in the codon context and use the residual values to further explore the matrices of residual values (see Statistics, above). The data generated are then converted into a contingency table that includes the corresponding observed values of Pearson's statistics, and the matrix of adjusted residuals [25].

After processing, the data become available to the visualization module. This module is the graphical interface. It follows the file manager paradigm in which information is presented in hierarchical views. This module offers a set of tools that enable several tasks to be carried out, namely to search prespecified sequence patterns, to visualize data in histogram form, to cluster codon context data, and to export residual values. It is also possible to visualize other information at the gene level, such as rare codons and their distribution in the ORFs, to determine their ratio relative to the total number of codons, to determine the GC% at the first, second and third codon positions and determine the codon adaptation index (CAI) and the effective number of codons [55,56].

## Figures and Tables

**Table 1:** The 3' codon context of CUG

3' Codon	Residual	3' Codon	Residual	3' Codon	Residual	3' Codon	Residual
AAA	7.436	ACG	0.644	UCU	-10.007	CCA	-2.438
AAG	1.927	CGU	-1.809	CUU	1.167	CCG	2.895
AAU	0.397	CGC	2.981	CUC	2.18	CAU	2.026
AAC	2.037	CGA	8.258	CUA	5.258	CAC	2.642
ACU	-6.947	CGG	5.404	CUG	6.774	CAA	4.049
ACC	-5.239	ACG	-4.726	CCU	-1.769	CAG	7.105
ACA	-5.12	AGG	-0.666	CCC	8.894	UAA	0.22

Positive values indicate that the 3' codons appear in the genome more times than expected (good context) while negative values indicate that the 3' codons appear fewer times than expected assuming a random distribution (bad context). Residual values give a quantitative indication of the context bias, where values falling within the -3 to +3 interval are not statistically significant (no bias). See also Figure 2.

**Table 2:** Ranking of the 10 most negative and 10 most positive residual values in *S. cerevisiae*, *S. pombe *and *C. albicans *contexts

*S. cerevisiae*		*S. pombe*		*C. albicans*	
Context	Residual	Context	Residual	Context	Residual

Most negative values					
**UUU → AAG**	**-24.58**	GAA → CCU	-24.159	UUU → CCA	-32.691
GAU → AAG	-22.487	GAU → AAG	-24.124	UUC → GAA	-31.586
AUU → AAA	-21.546	**UUU → AAG**	**-23.899**	UCA → GAU	-28.317
**AUU → AAG**	**-21.285**	AUU → AAA	-22.923	**AUU → AAG**	**-28.284**
CUU → AAA	-20.656	UCU → AAG	-22.334	GGU → UUU	-27.198
UUU → AAA	-20.563	CUU → AAA	-21.25	AAC → UUA	-26.198
UCC → GAA	-20.069	GUU → AAA	-21.218	GAC → UUA	-25.795
AAG → UCU	-19.706	**AUU → AAG**	**-21.08**	**UUU → AAG**	**-25.316**
GAU → CAA	-19.274	UUU → AAA	-20.704	GGA → AAA	-25.26
GAA → CCA	-19.155	GAA → UCU	-20.698	UUC → GAU	-24.822
					
Most positive values					
GAU → GAU	29.839	CAG → CAA	25.279	ACA → ACA	49.476
AAG → AAG	29.937	GAA → GAG	25.644	CAC → CAC	49.511
UUG → AAA	30.459	AAG → AAG	26.901	CCA → CCA	52.889
**GAA → GAA**	**30.573**	CUU → CGU	27.013	**GAA → GAA**	**57.356**
AAG → AAA	31.427	**GAA → GAA**	**28.051**	AAG → AAA	58.605
CAG → CAA	33.445	AGA → AGA	29.623	**GCU → GCU**	**62.611**
AGA → AGA	33.798	AAA → AAG	30.358	ACC → ACC	70.117
**GGU → GGU**	**35.979**	**GCU → GCU**	**32.158**	**GGU → GGU**	**72.48**
**GCU → GCU**	**36.231**	**GGU → GGU**	**33.681**	AAC → AAC	87.115
CAG → CAG	45.422	UCU → UCU	35.086	CAA → CAA	105.216

Anaconda was used to analyze the codon context of the complete genomes of *S. cerevisiae*, *S. pombe *and *C. albicans*. All possible codon contexts were ranked according to their calculated adjusted residuals, and the 10 most negative and 10 most positive were selected as extreme examples. The results indicate that only a small number of bad or good codon pairs (shown in bold) are shared between all three yeast species.

**Table 3:** Ranking of the codon pairs that display the highest residual difference between *S. cerevisiae*, *S. pombe *and *C. albicans*

*S. pombe-S. cerevisiae*		*S. pombe-C. albicans*		*C. albicans-S. cerevisiae*	
Context	Difference	Context	Difference	Context	Difference

CAG → CAG	27,798	**CAA → CAA**	**100,639**	**CAA → CAA**	**79,38**
UUG → AAA	25,266	AAC → AAC	76,716	AAC → AAC	62,939
CUU → CGU	25,168	ACC → ACC	60,208	ACC → ACC	50,735
CAA → CAG	24,507	CCA → CCA	47,603	CCA → CCA	39,196
AAA → AAG	23,593	ACA → ACA	47,359	CAC → CAC	39,032
UUC → AAA	22,86	CAC → CAC	47,175	ACA → ACA	39,029
AAU → AAU	22,021	GGA → AAA	45,043	GGU → GGU	36,501
**CAA → CAA**	**21,259**	AAG → AAA	43,994	GGA → UUA	35,81
GUU → CUU	21,194	CAA → CAG	43,927	GGA → AAA	29,786
GAU → GAC	19,483	UCA → UCA	41,533	GUU → GAU	29,753

Anaconda was used to analyze the codon context of the complete genomes of *S. cerevisiae*, *S. pombe *and *C. albicans*. The adjusted residuals of each codon context calculated for each pair of genomes - that is, *S. pombe-S. cerevisiae*; *S. pombe-C. albicans*; and *C. albicans-S. cerevisiae *- were subtracted and the result converted into a positive number by a module calculation. These values were used to rank the respective codon contexts and the 10 highest cases obtained were selected. Among these three yeast species, *S. pombe *and *S. cerevisiae *display the lowest differences, with the maximum value of the difference being found for the CAG-CAG pair (27.798). For *S. pombe *and *C. albicans *that value reaches 100.639 for the CAA-CAA codon pair. It is noteworthy that the highest difference value for the former pair is lower than the lowest value for the latter in this ranking of context differences. The only codon pair shared between all three yeast pairs is shown in bold.

**Table 4:** GC3 influences codon-pair context

	Residuals				
ORFeome	[- ∞, -9]	[-9, -3]	[-3, 3]	[3, 9]	[9, + ∞]

*S. cerevisiae*	0.0	2.5	**94.2**	3.3	0.0
*E. coli*	0.7	15.2	**67.1**	15.0	2.0

In order to measure the influence of GC bias on codon-pair context, the percentage of adjusted residuals that reversed their residual signals from positive to negative (or vice versa) between low and high GC3 subgroups of ORFs was determined. Most of the residual signal inversions for both species considered fall within the nonstatistically significant interval of the residuals (-3 to +3) indicating that GC3 bias is mainly felt in codon-pairs where the association is very weak or nonexistent (highlighted in bold).

**Table 5:** A hypothetical *r *× *c *contingency table

	B_1_	...	B_j_	...	B_c_	Marginal total
A_1_	n_11_	...	n_1j_	...	n_1c_	n_1_*
...		...		...		
A_l_	n_l1_	...	n_ij_	...	n_lc_	n_1_*
...		...		...		
A_r_	n_r1_	...	n_rl_	...	n_rc_	n_r_*
Marginal total	n*_1_	...	n*_i_	...	n*_c_	N

The table illustrates how contingency tables were constructed and how the statistical methodologies described in methods were implemented. One set of categories is represented by rows, the other by columns. In the present case, if the 3' context is being analyzed by Anaconda the rows of the table (A) correspond to the 5' codons and the columns (B) to the 3' codons of each pair.
